# Satisfaction-Behavior Paradox in Lifestyle Choices: A Cross-Sectional Study of Health Behaviors and Satisfaction Levels in Jazan, Saudi Arabia

**DOI:** 10.3390/healthcare12171770

**Published:** 2024-09-04

**Authors:** Anwar M. Makeen, Ibrahim M. Gosadi, Mohammad A. Jareebi, Mohammed A. Muaddi, Abdullah A. Alharbi, Ahmed A. Bahri, Majed A. Ryani, Mohamed Salih Mahfouz, Tariq Al Bahhawi, Ammar K. Alaswad, Talal A. Hamdi, Rahaf I. Qussadi, Aljazi A. Munhish, Ahmad Y. Alqassim

**Affiliations:** 1 Family and Community Medicine Department, Faculty of Medicine, Jazan University, Jazan 45142, Saudi Arabia; amakeen@jazanu.edu.sa (A.M.M.); mothman@jazanu.edu.sa (M.A.M.); aaalharbi@jazanu.edu.sa (A.A.A.); dr.bahri2010@gmail.com (A.A.B.); majedryani@gmail.com (M.A.R.); mm.mahfouz@gmail.com (M.S.M.); albahhawi@gmail.com (T.A.B.); aalqassim@jazanu.edu.sa (A.Y.A.); 2 Faculty of Medicine, Jazan University, Jazan 45142, Saudi Arabia; ammar.alaswad24@gmail.com (A.K.A.); tala.lha023@gmail.com (T.A.H.); rahafqussadi@gmail.com (R.I.Q.); aljaziabdu2004@gmail.com (A.A.M.)

**Keywords:** lifestyle, satisfaction, obesity, physical activity, diet, health behavior, public health, environmental health, Saudi Arabia

## Abstract

Background: Lifestyle choices significantly affect health outcomes in Saudi Arabia, but the relationship between health behaviors and satisfaction is unclear. This study aimed to assess lifestyle choices and associated satisfaction levels among residents in the Jazan region of Saudi Arabia. Methods: A cross-sectional study was conducted in Jazan, Saudi Arabia, with 3411 participants. Sociodemographic, lifestyle, and satisfaction data were collected using a questionnaire that was completed during interviews. Logistic regression analyses were implemented to examine the relationships between the study variables and factors related to weight, physical activity, and eating satisfaction. Results: The study found that 38.3% of participants were inactive and 77% rarely ate fruits and vegetables. A total of 47.6% were overweight or obese. Weight (71.12%) and eating (71.59%) satisfaction were high despite these health concerns. The satisfaction-behavior paradox was especially evident in diet. Higher income, physical activity, and healthy eating habits were significantly associated with higher satisfaction (OR = 1.49, 95% CI: 1.15–1.93, *p* = 0.003 for weight satisfaction, OR = 34.74, 95% CI: 25.42–48.35, *p* < 0.001 for physical activity satisfaction, and OR = 2.08, 95% CI: 1.67–2.61, *p* < 0.001 for fruit and vegetable consumption). Conclusions: Lifestyle behaviors and satisfaction in Jazan, Saudi Arabia, are complex. The satisfaction-behavior paradox, especially in diet, reveals a major gap between perceived and actual health. These findings highlight the need for targeted, culturally sensitive interventions that address behavioral change and perception alignment to improve regional public health.

## 1. Introduction

Global health outcomes and well-being depend more on lifestyle choices and health behaviors. The World Health Organization defines a healthy lifestyle as “a way of living based on identifiable patterns of behavior which are determined by the interplay between an individual’s personal characteristics, social interactions, and socioeconomic and environmental living conditions” [1]. This definition encompasses various aspects of daily life including diet, physical activity, tobacco use, and alcohol consumption [2].

Numerous studies have demonstrated the positive impact of healthy lifestyles on reducing the chronic disease risk and improving health management. For instance, the United States Diabetes Prevention Program, a randomized clinical trial multi-center, has investigated the effect of lifestyle intervention on diabetes risk [3]. The program achieved a 58% reduction in the diabetes incidence rate by setting goals of reducing body weight by 7% and maintaining a physical activity level of 700 calories per week [3]. This study’s findings are particularly relevant to Saudi Arabia given the country’s high prevalence of diabetes. According to recent estimates, approximately 17.7% of the adult Saudi population was affected by diabetes in 2021 [4], highlighting the urgent need for effective lifestyle interventions. More recent studies have confirmed the health benefits of lifestyle interventions that have yielded significant reductions in body weight, body mass index (BMI), and waist circumference among adults with metabolic syndrome [5,6].

While the importance of healthy lifestyles is universally recognized, their implementation and maintenance vary across cultures and socioeconomic groups. This variability points to the need for targeted healthy lifestyle promotion in diverse populations. In this context, Saudi Arabia presents a unique case study of how rapidly developing nations must manage the health effects of urbanization and lifestyle changes. According to previous studies, the main lifestyle issues in Saudi Arabia are inactivity, poor diet, tobacco use, and stress [7]. A systematic review revealed that Saudi Arabia has a higher obesity rate than the global average, with the largest studies reporting a prevalence of 35.6%. Obesity in Saudi Arabia is primarily associated with dietary factors (particularly the shift toward the Western diet and increased consumption of sugary beverages), physical inactivity, and demographic factors such as being female, married, and living in an urban area [8]. There has been a significant rise in chronic illnesses in Saudi Arabia associated with urbanization and the adoption of Western lifestyles. Sedentary lifestyles combined with a greater consumption of fast food have increased the risk of obesity [9,10,11,12]. In the World Health Report of Saudi Arabia 2019, which used a participant sample of 8912, only 39% of the respondents had a normal BMI, and nearly 60% of adult Saudis were either overweight or obese [13]. These alarming statistics highlight the need for effective public health interventions nationwide.

The Saudi government has promoted healthy lifestyles and physical activity to address the growing problem of obesity in the nation [14]. Recent initiatives include the Quality of Life Program, which aims to increase the percentage of individuals who exercise at least once a week from 13% in 2015 to 40% by 2030 [15]. These programs have raised the awareness of healthy lifestyles and led to the construction of more public recreational facilities, but they have also encountered challenges including cultural barriers to women’s public physical activity and a lack of comprehensive health education programs [16]. A recent study found that behavioral changes were difficult to implement despite increased awareness, highlighting the complexity of population-level lifestyle modification [16]. Initiatives that seek to address these challenges and adapt strategically to Saudi Arabia’s unique cultural context are most likely to succeed.

Research on lifestyle-related health issues in Saudi Arabia is limited due to a lack of understanding of the factors influencing an individual’s satisfaction with their lifestyle choices, a focus on urban populations, and the interaction between lifestyle behaviors and socioeconomic factors [17]. Understanding these gaps can aid in the design of effective public health interventions. Theoretical frameworks that assess lifestyle behaviors and change readiness are needed to address the current limitations and develop more targeted interventions.

Assessing the community lifestyle risk factors is crucial to designing effective public health interventions. Such assessments should also consider the community members’ willingness to change unhealthy habits. The transtheoretical model, which measures lifestyle change motivation, is useful in this context. This model describes six stages of behavioral change, from pre-contemplation to maintenance [18]. Interestingly, research suggests that some high-risk individuals may enjoy their unhealthy lifestyles. Satisfaction may make it harder to change unhealthy habits. A recent study in Saudi Arabia found that individuals who reported high satisfaction with their current lifestyle, despite engaging in unhealthy behaviors, were less likely to participate in health promotion programs [17]. This finding underscores the importance of understanding satisfaction levels when designing health interventions.

Given these considerations, the current study aimed to assess lifestyle choices among individuals in a community from the southwestern region of Saudi Arabia and identify factors associated with levels of satisfaction. The study’s findings may significantly impact public health policy in Saudi Arabia by elucidating the relationship between lifestyle behaviors, satisfaction levels, and behavioral change. The conclusions of the present study may be used to guide the design of culturally sensitive health programs, allocate resources, and reduce lifestyle-related diseases. The research may also serve as a model for other developing nations.

## 2. Methods

### 2.1. Study Design and Setting

This cross-sectional study was part of a regional health needs assessment project conducted in May and June 2024. Cross-sectional designs are ideal for capturing population health behaviors and attitudes at a specific time [19]. The researchers were able to efficiently collect data on multiple variables simultaneously, providing a comprehensive picture of Jazan’s lifestyle patterns and satisfaction levels. The study was conducted in the Jazan region of Saudi Arabia, a diverse area with a rich cultural heritage and rapid urbanization. The region’s proximity to the Yemeni border and its transition from an agricultural economy to an urban economy provide an interesting context for examining lifestyle choices and satisfaction levels across socioeconomic groups.

### 2.2. Data Collection Tool

The health needs assessment tool was developed through a rigorous process involving consultation of the relevant international literature on community health needs assessment tools [20,21] and was reviewed by a panel of experts in family and community medicine to ensure its comprehensiveness and suitability for the target population. The final questionnaire comprised various components including demographics, lifestyle determinants of health, and the participants’ satisfaction with their lifestyles. The lifestyle determinants assessed in this study were based on the Saudi Guidelines for the Prevention and Management of Obesity [22]. Participants self-reported their body weight and height, allowing for BMI calculation. Physical activity levels were evaluated by asking participants about their adherence to the clinical recommendation of performing 150 min or more of exercise per week. Dietary behaviors were assessed through questions on five key elements of healthy eating designed to measure the participants’ consumption of whole-grain products, fruits and vegetables, low-fat meats, and low-fat products, along with their avoidance of high-sugar foods. Satisfaction with body weight, physical activity, and eating behavior was measured using a Likert scale ranging from 1 (lowest satisfaction) to 10 (highest satisfaction) for each parameter. The internal consistency of the lifestyle determinants and satisfaction items was evaluated using Cronbach’s alpha, yielding a coefficient of 0.77, indicating acceptable reliability.

### 2.3. Data Collection Process

Trained medical students conducted personal interviews to collect data. The students received two days of training on interviewing, ethics, and questionnaire use. To ensure the consistency and accuracy of the data collection, the training included role-playing and practice interviews. Quality control was used throughout the data collection. Daily supervisor debriefings, random questionnaire spot checks, and 5% participant re-interviews to assess reliability were incorporated. Discrepancies were resolved quickly. Community settings in various Jazan provinces hosted the interviews. Participants were identified and approached in their communities. This household survey recruited in the targeted individuals’ homes. Adults (18+) and Jazan residents were eligible. Non-Jazan residents and minors were excluded. In addition, individuals who were identified and approached but who declined to participate were excluded.

Multistage sampling was used to ensure regional representation. The Jazan region, which is composed of 17 governmental provinces in coastal, mountain, and island areas, was first stratified by geography. Districts and households were randomly selected within each stratum. Parents and children from each household were selected to gather intergenerational perspectives.

### 2.4. Study Size

Sample size estimation of the current health needs assessment was based on the statistical formula:

n_h_ = (*deff*) (Z^2^) (P)(1 − P) (k)/(Ã)(d^2^),

where

n_h_ is the sample size in terms of the number of households to be selected;

*deff* is the sample design effect;

*z* is the statistic that defines the level of confidence desired;

*P* is an estimate of a key indicator to be measured by the survey;

*k* is a multiplier to account for the anticipated rate of non-response;

Ã Average no. of persons of the base population per HH;

*d* is the margin of error to be attained.

In the survey, the number of 1600 households was estimated based on the following calculations. First, the z-statistic = 1.96 for the 95% level of confidence. The default value of the sample design effect was set as 2, unless there was supporting empirical data from previous or related surveys that suggested a different value. For the non-response multiplier, *k*, a proportion of 20% was suggested. The level of P was set as 50% because we were interested in many health indicators, so it was best to use this percentage as it provided the largest sample size. Finally, the average no. of persons of the base population (above 18 years) per HH was 60%.

This approach allowed for the comprehensive assessment of lifestyle choices and satisfaction levels across diverse geographic and demographic segments of the Jazan population, enhancing the generalizability of the findings to the broader community [17,23].

### 2.5. Data Analysis

Data analysis was performed using R software (version 4.2.3, R Foundation for Statistical Computing, Vienna, Austria). The risk factors examined were divided into two main categories. The first category included sociodemographic characteristics such as age, gender, education, residence, income, nationality, languages spoken, employment status, marital status, and housing. The second category focused on health-related parameters including height, weight, BMI, physical activity levels, smoking habits, khat usage, and dietary behaviors. The outcome variables of interest were satisfaction levels regarding weight, physical activity, and dietary behaviors among the study participants.

Our analysis began with an overview of the sample characteristics. Binary and categorical variables were described using frequencies and proportions, and quantitative variables were summarized with mean values and standard deviations (SDs). Additionally, we used multiple logistic regression analyses to investigate the adjusted relationships between the outcome variables and the identified risk factors, quantifying these associations with the odds ratios (ORs). Statistical significance was determined with a *p*-value threshold below 0.05.

## 3. Results

### 3.1. Sociodemographic, Habitual, and Dietary Characteristics of the Sample

Table 1 describes the socio-demographic characteristics of 3411 participants from the Jazan region in Saudi Arabia. The mean age was 34 ± 15 years, with households averaging 7 ± 2.9 family members. This study recruited a nearly equal sample of male and female participants (50.9% vs. 49.1%, respectively). The majority of participants were Saudi nationals (97%) and Arabic speakers (99.7%). Most participants were university-educated (54.3%), single (61%), and resided in rural areas (62%). A significant portion were students (37.6%), with the highest income bracket being ≥15,000 SAR (27.7%). Housing was predominantly in owned villas (46%). Table 2 presents the health characteristics of the study population. The mean height was 163 ± 9.3 cm, the mean weight was 67 ± 17 kg, and the mean BMI was 25 ± 5.6. Regarding physical activity, 38.3% of participants reported no physical activity per week, while 40.3% engaged in moderate or vigorous activity for a minimum of 30 minutes, five times per week, which was the highest reported activity level. BMI categories indicated that 41.3% of participants were of normal weight, 30.1% were overweight, 17.5% were obese, and 11.1% were underweight. Table 3 describes the smoking behavior and khat chewing habits of the study participants. The majority (81%) reported never smoking. Meanwhile, 10% were current smokers, and 4% were ex-smokers. Passive smoking was reported by 5% of participants. In terms of specific smoking types, 91% were non-cigarette smokers, 95% were non-shisha smokers, and 96% were non-vape smokers. The prevalence of combined smoking behaviors was generally low, with 1% or less engaging in combinations of cigarettes, shisha, and vape smoking. Regarding khat chewing, 89.7% reported never chewing khat, 4.7% were current users, and 5.5% were ex-users. Table 4 presents the dietary habits of the study participants (n = 3411). Approximately half of all participants reported consuming whole grains (48%), while a slightly higher percentage reported not consuming whole grains (52%). Daily consumption of fruits and vegetables was reported by 23% of participants, with the majority (77%) indicating infrequent consumption. Regarding meat choices, 51% reported consuming low-fat meats, while 49% did not. A significant proportion of participants reported avoiding high-sugar foods (37%) and consuming low-fat products (32%), although the majority did not engage in these dietary practices (63% and 68%, respectively).

### 3.2. Satisfaction Levels among Participants

**Figure 1:**
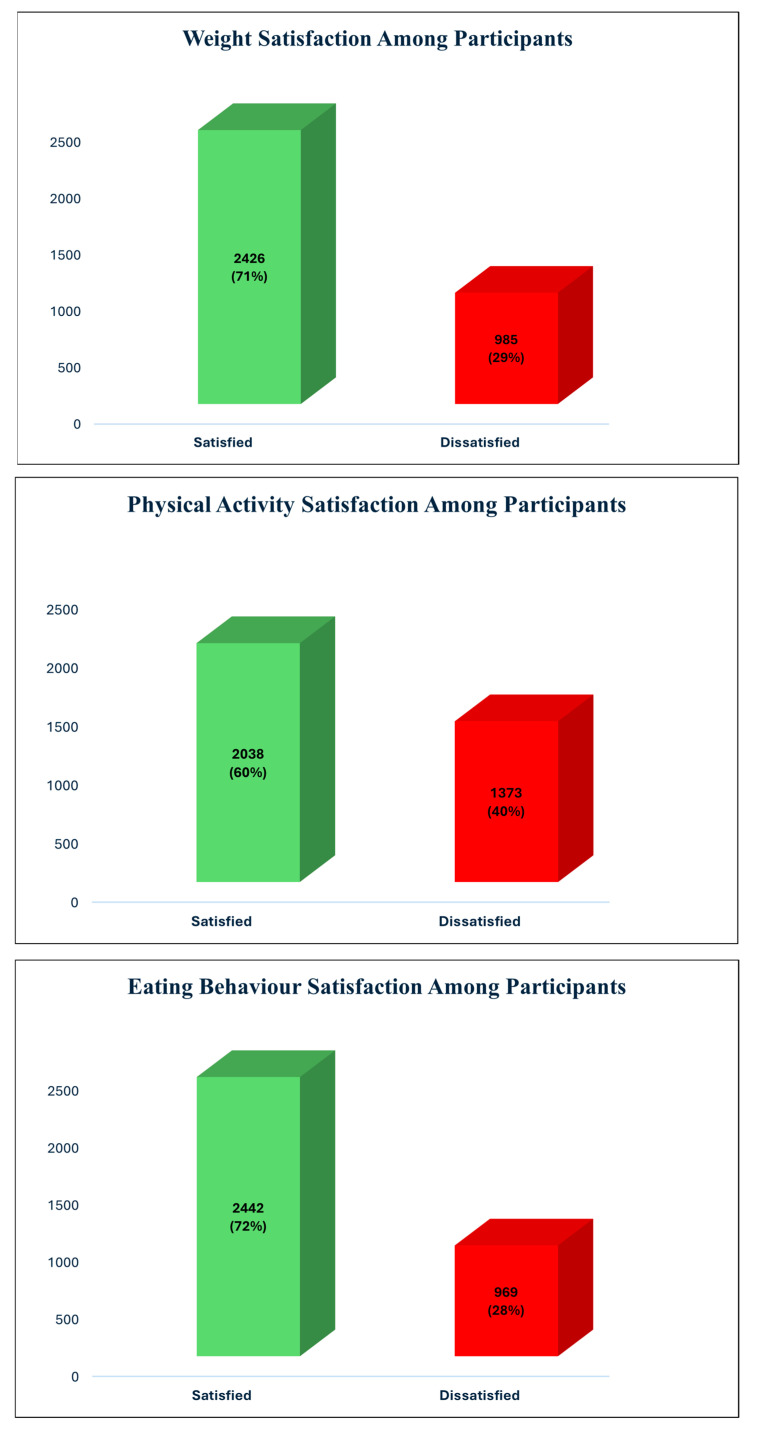
Percentage distribution of satisfaction levels for weight, physical activity, and eating behavior among participants in Jazan region, Saudi Arabia (n = 3411).

The majority of participants were satisfied with their weight and eating behaviors. Specifically, 71.12% of participants reported satisfaction with their weight, while 28.88% were dissatisfied. In terms of eating behavior, 71.59% were satisfied, and 28.41% were dissatisfied. However, satisfaction with physical activity was relatively lower, with 59.75% of the participants expressing satisfaction and 40.25% indicating dissatisfaction (Figure 1).

### 3.3. The Determinants of Satisfaction Levels among Participants

Table 5 shows the association of demographic and lifestyle factors with weight satisfaction among the participants. There was a significant association between nationality and weight satisfaction, with Saudi nationals being less likely to be satisfied compared with other nationalities (OR = 0.54, CI = 0.28–0.98, *p* = 0.049). Income level also played a significant role: participants earning between 10,000 and 14,999 SAR were more likely to be satisfied with their weight compared with those earning less than 5000 SAR (OR = 1.49, CI = 1.15–1.93, *p* = 0.003). Housing type was another significant factor, with participants living in owned villas being less likely to be satisfied with their weight compared with those living in rented housing (OR = 0.66, CI = 0.49–0.89, *p* = 0.007). BMI showed a significant negative association with weight satisfaction (OR = 0.89, CI = 0.84–0.94, *p* < 0.001). Additionally, BMI categories indicated that underweight (OR = 0.18, CI = 0.12–0.27, *p* < 0.001), overweight (OR = 0.45, CI = 0.32–0.64, *p* < 0.001), and obese individuals (OR = 0.28, CI = 0.15–0.50, *p* < 0.001) were all less likely to be satisfied with their weight compared with those of a normal weight. Physical activity levels showed significant associations, with participants engaging in mild-to-moderate (OR = 1.48, CI = 1.22–1.79, *p* < 0.001) and moderate-to-vigorous physical activity (OR = 1.87, CI = 1.46–2.39, *p* < 0.001) being more likely to be satisfied with their weight compared with those with no physical activity. Finally, eating behavior related to the consumption of low-fat meat was significantly associated with weight satisfaction, with participants choosing low-fat meat being more likely to be satisfied with their weight (OR = 1.33, CI = 1.12–1.58, *p* = 0.001).

Table 6 shows the association of demographic and lifestyle factors with physical activity satisfaction among participants. Nationality was significantly associated with physical activity satisfaction, with Saudi nationals being less likely to be satisfied compared with other nationalities (OR = 0.51, CI = 0.28–0.90, *p* = 0.022). Physical activity levels had a strong association, where participants engaging in mild-to-moderate physical activity (OR = 7.23, CI = 6.03–8.71, *p* < 0.001) and moderate-to-vigorous physical activity (OR = 34.74, CI = 25.42–48.35, *p* < 0.001) were significantly more likely to be satisfied compared with those with no physical activity. Eating behaviors also played a significant role: participants who chose low-fat meat (OR = 1.32, CI = 1.11–1.57, *p* = 0.002), avoided high-sugar foods (OR = 1.27, CI = 1.06–1.52, *p* = 0.008), and opted for low-fat products (OR = 1.25, CI = 1.03–1.50, *p* = 0.021) were more likely to be satisfied with their physical activity levels than those who did not make these choices.

Table 7 shows the association of demographic and lifestyle factors with eating behavior satisfaction among the participants. Age was a significant factor, with older participants being more satisfied with their eating behavior (OR = 1.02, CI = 1.01–1.04, *p* < 0.001). Males were more likely to be satisfied compared with females (OR = 1.27, CI = 1.05–1.54, *p* = 0.015). Saudi nationals were less likely to be satisfied compared with individuals of other nationalities (OR = 0.35, CI = 0.17–0.65, *p* = 0.002). BMI was inversely related to satisfaction (OR = 0.92, CI = 0.87–0.96, *p* = 0.001), with underweight participants reporting significantly less satisfaction with their eating behaviors compared with those with normal weight (OR = 0.53, CI = 0.37–0.76, *p* = 0.001).

Physical activity levels were significantly associated with eating behavior satisfaction, where participants engaging in mild-to-moderate (OR = 1.93, CI = 1.61–2.32, *p* < 0.001) and moderate-to-vigorous physical activity (OR = 2.68, CI = 2.10–3.44, *p* < 0.001) were more likely to be satisfied compared with those with no physical activity. Various eating behaviors were also significant: participants consuming daily fruits and vegetables (OR = 2.08, CI = 1.67–2.61, *p* < 0.001), choosing low-fat meat (OR = 1.90, CI = 1.61–2.25, *p* < 0.001), avoiding high-sugar foods (OR = 1.83, CI = 1.53–2.20, *p* < 0.001), and opting for low-fat products (OR = 2.02, CI = 1.67–2.46, *p* < 0.001) were more likely to be satisfied with their eating behaviors than participants who did not make these choices.

## 4. Discussion

The present study revealed significant insights into the lifestyle choices and satisfaction levels of residents of the Jazan region in Saudi Arabia. Our findings highlight a complex relationship between health behaviors and satisfaction, particularly in the domains of physical activity, diet, and weight management. Notably, we observed a paradoxical trend where high levels of satisfaction coexisted with potentially unhealthy lifestyle choices.

A notable finding was the high prevalence of physical inactivity, with over a third of participants reporting no regular exercise. This is concerning given the well-established benefits of physical activity in preventing chronic diseases. Similarly, dietary habits showed room for improvement, with most participants reporting the infrequent consumption of fruits and vegetables. Despite these unhealthy behaviors, satisfaction levels regarding weight and eating habits were surprisingly high. This discrepancy between actual health behaviors and perceived satisfaction suggests a potential misalignment between health awareness and practice in the community.

A remarkable paradox was found between unhealthy lifestyle behaviors and high satisfaction, particularly in diet. This satisfaction-behavior paradox suggests that many students are unaware of their problematic habits and are in the precontemplation stage of behavior change [18]. In dietary behaviors, satisfaction levels were similar regardless of healthy eating guidelines, highlighting the disconnect. This differed from the patterns of satisfaction noted for weight and physical activity levels. Several factors may be responsible for this paradox. For example, Saudi Arabian culture emphasizes social eating and high-calorie dishes, which may normalize unhealthy diets [9]. A lack of health awareness and nutritional literacy among students may also lead to misconceptions about healthy diets [23]. Cognitive dissonance may also play a contributing role, with students rationalizing unhealthy behaviors to boost their self-esteem [11]. Health education and behavior change interventions in the region should take heed of this paradox. If students are happy with unhealthy behaviors, traditional education may be ineffective. Instead, interventions should raise awareness of the discrepancy between current behaviors and health recommendations, possibly using motivational interviewing [8]. Addressing the environmental and cultural factors that underlie this paradox, such as campus food availability and social norms of physical activity, may help promote behavioral change [7,16].

Our findings indicate that targeted public health interventions and policies are needed to address Jazan’s high prevalence of unhealthy lifestyle behaviors and paradoxical satisfaction levels. Multifaceted interventions should address individual, environmental, and sociocultural factors [9]. Policies that make healthy foods more accessible and affordable, along with nutrition education, can improve diets [8]. Community-based, culturally appropriate physical activity programs, especially for women, can be implemented to address inactivity [16]. Given the satisfaction paradox, especially in dietary behaviors, health promotion programs should highlight the gap between perceived and actual health [17]. The transtheoretical model of behavior change [18] may influence motivational interviewing and stage-based interventions. University policies, such as offering healthy food in cafeterias and increasing campus physical activity, could encourage healthier choices [16]. The engagement of community leaders and social networks may improve the cultural sensitivity and effectiveness of these interventions [7]. Finally, tailored substance abuse prevention programs should be integrated into health promotion efforts to address regional issues such as khat use [9]. Public health initiatives can better address the complex interactions that affect the lifestyle behaviors and satisfaction of the residents of Jazan by implementing these comprehensive, culturally sensitive strategies.

### Strengths and Limitations

This study has several strengths including its large sample size, its use of a culturally relevant and validated questionnaire, and its comprehensive assessment of various lifestyle factors. Robust statistical analyses illuminate the complex relationships between sociodemographic factors, lifestyle behaviors, and satisfaction. However, some restrictions remain. Cross-sectional studies cannot establish causality. Self-reported data from direct interviews may be biased, especially on sensitive topics such as diet and exercise. Results from this single-region sample may not apply to the entire national population. In addition, cultural and environmental factors specific to Jazan may affect the lifestyle choices and perceptions differently. Despite these limitations, this study provides a solid foundation for understanding the complex relationship between lifestyle behaviors and satisfaction in Saudi Arabia, which can inform targeted public health interventions.

## 5. Conclusions

This study examined lifestyle behaviors and satisfaction in Jazan, Saudi Arabia, revealing a complex relationship between health behaviors, sociodemographic factors, and satisfaction. The findings showed a worrying prevalence of unhealthy lifestyle behaviors including inactivity, poor diet, and obesity. The most surprising finding was the satisfaction-behavior paradox, where participants reported high health satisfaction despite engaging in unhealthy dietary habits. This paradox highlights a critical gap between perceived and actual health status, making public health interventions more challenging. We found significant associations between sociodemographic factors and satisfaction, emphasizing the need for culturally sensitive interventions. The results of this study underscore the need to address the environmental and sociocultural factors that lead to unhealthy lifestyles and call for a health promotion paradigm shift. Addressing this disconnect and implementing targeted, multilevel interventions that consider individual behaviors, community norms, and societal structures could significantly improve regional health outcomes. Longitudinal studies should examine the temporal relationships between health behaviors, satisfaction, and long-term health outcomes and the efficacy of interventions to address the satisfaction-behavior paradox.

## Figures and Tables

**Table 1:** Sociodemographic characteristics of the study participants (n = 3411).

Characteristics	Mean ± SD
Age	34 ± 15 years
Family member	7 (5–8) * kids/household
**Characteristics**	**Frequency (%)**
Gender	
Male	1735 (50.9%)
Female	1676 (49.1%)
Nationality	
Saudi	3312 (97%)
Non-Saudi	99 (3%)
Language	
Arabic	3401 (99.7%)
Other	10 (0.3%)
Education	
Illiterate	87 (2.5%)
Elementary	96 (2.8%)
Intermediate	119 (3.5%)
Secondary	1135 (33.3%)
University	1852 (54.3%)
Postgraduate	122 (3.6%)
Marital status	
Single	1678 (49%)
Married	1591 (47%)
Divorced	70 (2%)
Widowed	72 (2%)
Divorced/widowed	142 (4%)
Employment	
Governmental sector employee	833 (24.4%)
Military employee	150 (4.4%)
Private sector employee	238 (7%)
Business owner	52 (1.5%)
Retiree	241 (7.1%)
Student	1281 (37.6%)
Housewife/houseman	375 (11%)
Unemployed	241 (7.1%)
Income	
“Less than 5000”	1083 (31.7%)
“5000–9999”	639 (18.7%)
“10,000–14,999”	747 (21.9%)
“≥15,000”	942 (27.7%)
Residence	
Rural	2099 (62%)
Urban	1312 (38%)
Housing	
Owned apartment	680 (20%)
Owned-traditional house	736 (22%)
Owned villa	1567 (46%)
Rented	428 (13%)

Abbreviations: SD—standard deviation, n—sample size. * Median (interquartile range: IQR).

**Table 2:** Physical activity patterns and anthropometric measures of study participants (n = 3411).

Characteristics	Mean ± SD
Height	163 ± 9.3 cm
Weight	67 ± 17 kg
BMI	25 ± 5.6
**Characteristics**	**Frequency (%)**
Physical activity	
No physical activity is performed per week	1305 (38.3%)
Moderate or vigorous activity for a minimum of 30 minutes, 5 times per week	1375 (40.3%)
Moderate or vigorous activity for a less than 30 minutes, 5 times per week	731 (21.4%)
BMI Categories	
Underweight	379 (11.1%)
Normal weight	1408 (41.3%)
Overweight	1027 (30.1%)
Obese	597 (17.5%)
BMI Categories (detailed)	
Underweight	379 (11.1%)
Normal weight	1408 (41.3%)
Overweight	1027 (30.1%)
Obese class I	428 (12.55%)
Obese class II	125 (3.66%)
Obese class III	44 (1.3%)

Abbreviations: SD—standard deviation, n—sample size.

**Table 3:** Smoking behavior and khat chewing habits among study participants (n = 3411).

Characteristics	Frequency (%)
Smoking behavior	
Never	2758 (81%)
Ex-smoker	150 (4%)
Current	334 (10%)
Passive	169 (5%)
Cigarettes smokers	
No	3116 (91%)
Yes	295 (9%)
Shisha smokers	
No	3226 (95%)
Yes	185 (5%)
Vape smokers	
No	3290 (96%)
Yes	121 (4%)
Cigarettes and shisha smokers	
No	3366 (99%)
Yes	45 (1%)
Cigarettes and vape smokers	
No	3387 (99%)
Yes	24 (1%)
Shisha and vape smokers	
No	3378 (99%)
Yes	33 (1%)
All smokers (cigarettes, shisha, and vape)	
No	3395 (99.5%)
Yes	16 (0.5%)
Khat chewing	
Never	3061 (89.7%)
Ex-user	188 (5.5%)
Current	162 (4.7%)

Abbreviations: SD—standard deviation, n—sample size.

**Table 4:** Dietary habits of study participants (n = 3411).

Characteristics	Frequency (%)
Eating Behavior: whole grain	
No	1789 (52%)
Yes	1622 (48%)
Eating Behavior: daily fruits and vegetables	
No	2640 (77%)
Yes	771 (23%)
Eating Behavior: low fat meat	
No	1685 (49%)
Yes	1726 (51%)
Eating Behavior: avoidance high sugar food	
No	2155 (63%)
Yes	1256 (37%)
Eating Behavior: low fat products	
No	2303 (68%)
Yes	1108 (32%)

Abbreviations: SD—standard deviation, n—sample size.

**Table 5:** Demographic and lifestyle factors associated with weight satisfaction: results from multiple logistic regression analysis.

Predictors	Weight Satisfaction		
	Odds Ratios	CI	*p*
Age	1.01	1.00–1.03	0.049
Gender [reference: female]			
[Male]	0.98	0.81–1.20	0.868
Nationality [reference: other nationalities]			
[Saudi]	0.54	0.28–0.98	0.049
Language [reference: Arabic language]			
[Other Languages]	1.04	0.23–7.34	0.965
Education [reference: illiterate]			
[Elementary]	1.04	0.50–2.16	0.917
[Intermediate]	1.67	0.79–3.54	0.182
[Secondary]	1.20	0.62–2.31	0.589
[Bachelor’s degree]	1.26	0.65–2.43	0.488
[Postgraduate]	1.24	0.55–2.81	0.599
Employment [reference: unemployed]			
[Governmental sector employee]	0.92	0.60–1.41	0.715
[Military employee]	0.84	0.48–1.48	0.542
[Private sector employee]	1.09	0.68–1.74	0.729
[Business owner]	0.74	0.36–1.61	0.440
[Retiree]	1.13	0.64–1.99	0.679
[Student]	1.01	0.69–1.47	0.942
[Housewife/houseman]	1.26	0.79–2.00	0.334
Marital status [reference: single]			
[Married]	0.92	0.67–1.27	0.624
[Divorced]	1.09	0.57–2.12	0.802
[Widowed]	1.38	0.67–2.89	0.386
Income [reference: less than 5000]			
[5000 to 9999 SAR]	1.30	1.00–1.69	0.052
[10,000–14,999 SAR]	1.49	1.15–1.93	0.003
[15,000 and above]	1.25	0.98–1.60	0.077
Residence [reference: rural]			
[Urban]	0.90	0.75–1.08	0.255
Housing [reference: rented]			
[Owned apartment]	0.79	0.57–1.09	0.157
[Owned-traditional]	0.84	0.60–1.16	0.292
[Owned villa]	0.66	0.49–0.89	0.007
BMI	0.89	0.84–0.94	<0.001
BMI CAT [reference: normal weight]			
[Underweight]	0.18	0.12–0.27	<0.001
[Overweight]	0.45	0.32–0.64	<0.001
[Obese]	0.28	0.15–0.50	<0.001
Smoking [reference: never]			
[Current smoker]	1.25	0.92–1.71	0.152
[Ex-smoker]	0.83	0.55–1.26	0.368
[Passive smoker]	0.79	0.55–1.15	0.216
Physical activity [reference: no physical activity]			
[mild to moderate]	1.48	1.22–1.79	<0.001
[moderate to vigorous]	1.87	1.46–2.39	<0.001
Eating Behavior: whole grain			
[Yes]	0.85	0.72–1.01	0.068
Eating Behavior: daily fruits and vegetables			
[Yes]	1.17	0.96–1.45	0.128
Eating Behavior: low fat meat			
[Yes]	1.33	1.12–1.58	0.001
Eating Behavior: avoidance high sugar food			
[Yes]	1.20	1.00–1.43	0.051
Eating Behavior: low fat products			
[Yes]	1.13	0.94–1.36	0.201
Observations	3411		
R^2^	0.20		

**Table 6:** Multivariate logistic regression analysis of factors associated with physical activity satisfaction among participants in Jazan region, Saudi Arabia (n = 3411).

Predictors	Physical Activity Satisfaction		
	Odds Ratios	CI	*p*
Age	1.01	1.00–1.02	0.137
Gender [reference: female]			
[Male]	1.01	0.83–1.23	0.906
Nationality [reference: other nationalities]			
[Saudi]	0.51	0.28–0.90	0.022
Language [reference: Arabic language]			
[Other Languages]	0.40	0.08–2.10	0.276
Education [reference: illiterate]			
[Elementary]	1.27	0.63–2.57	0.506
[Intermediate]	1.36	0.66–2.79	0.403
[Secondary]	0.98	0.52–1.85	0.945
[Bachelor’s degree]	0.78	0.42–1.47	0.444
[Postgraduate]	0.59	0.27–1.29	0.186
Employment [reference: unemployed]			
[Governmental sector employee]	0.93	0.61–1.41	0.727
[Military employee]	1.03	0.57–1.87	0.927
[Private sector employee]	1.03	0.64–1.66	0.892
[Business owner]	0.56	0.27–1.21	0.134
[Retiree]	0.74	0.42–1.28	0.277
[Student]	0.71	0.48–1.03	0.071
[Housewife/houseman]	0.98	0.62–1.56	0.945
Marital status [reference: single]			
[Married]	1.09	0.79–1.51	0.597
[Divorced]	0.70	0.37–1.33	0.279
[Widowed]	0.95	0.47–1.91	0.876
Income [reference: less than 5000]			
[5000 to 9999 SAR]	1.29	0.99–1.68	0.060
[10,000–14,999 SAR]	1.02	0.79–1.31	0.889
[15,000 and above]	1.03	0.80–1.32	0.810
Residence [reference: rural]			
[Urban]	0.96	0.80–1.15	0.628
Housing [reference: rented]			
[Owned apartment]	0.85	0.62–1.16	0.299
[Owned-traditional]	1.12	0.81–1.56	0.485
[Owned villa]	0.92	0.69–1.23	0.574
BMI	0.96	0.91–1.01	0.153
BMI CAT [reference: normal weight]			
[Underweight]	1.16	0.79–1.72	0.445
[Overweight]	0.79	0.56–1.10	0.167
[Obese]	0.80	0.45–1.41	0.435
Smoking [reference: never]			
[Current smoker]	1.26	0.92–1.71	0.146
[Ex-smoker]	1.23	0.79–1.91	0.365
[Passive smoker]	0.86	0.59–1.27	0.452
Physical activity [reference: no physical activity]			
[mild to moderate]	7.23	6.03–8.71	<0.001
[moderate to vigorous]	34.74	25.42–48.35	<0.001
Eating Behavior: whole grain			
[Yes]	0.86	0.72–1.02	0.084
Eating Behavior: daily fruits and vegetables			
[Yes]	1.20	0.98–1.48	0.078
Eating Behavior: low fat meat			
[Yes]	1.32	1.11–1.57	0.002
Eating Behavior: avoidance high sugar food			
[Yes]	1.27	1.06–1.52	0.008
Eating Behavior: low fat products			
[Yes]	1.25	1.03–1.50	0.021
Observations	3411		
R^2^	0.32		

**Table 7:** Multivariate logistic regression analysis of factors associated with eating behavior satisfaction among participants in Jazan region, Saudi Arabia (n = 3411).

Predictors	Eating Behavior Satisfaction		
	Odds Ratios	CI	*p*
Age	1.02	1.01–1.04	<0.001
Gender [reference: female]			
[Male]	1.27	1.05–1.54	0.015
Nationality [reference: other nationalities]			
[Saudi]	0.35	0.17–0.65	0.002
Language [reference: Arabic language]			
[Other Languages]	0.82	0.18–4.38	0.802
Education [reference: illiterate]			
[Elementary]	1.30	0.55–3.05	0.547
[Intermediate]	1.00	0.44–2.25	0.992
[Secondary]	0.76	0.36–1.56	0.469
[Bachelor’s degree]	0.70	0.33–1.41	0.328
[Postgraduate]	0.50	0.21–1.17	0.114
Employment [reference: unemployed]			
[Governmental sector employee]	0.97	0.65–1.46	0.889
[Military employee]	0.84	0.48–1.48	0.548
[Private sector employee]	1.02	0.66–1.60	0.918
[Business owner]	0.73	0.35–1.59	0.410
[Retiree]	1.31	0.72–2.39	0.381
[Student]	1.19	0.84–1.69	0.326
[Housewife/houseman]	1.23	0.78–1.93	0.376
Marital status [reference: single]			
[Married]	1.07	0.78–1.45	0.680
[Divorced]	0.89	0.47–1.69	0.707
[Widowed]	0.65	0.32–1.33	0.230
Income [reference: less than 5000]			
[5000 to 9999 SR]	1.18	0.92–1.53	0.201
[10,000–14,999 SR]	1.15	0.90–1.47	0.275
[15,000 and above]	1.15	0.91–1.46	0.246
Residence [reference: rural]			
[Urban]	1.03	0.87–1.23	0.705
Housing [reference: rented]			
[Owned apartment]	1.06	0.78–1.44	0.696
[Owned-traditional]	1.03	0.75–1.41	0.859
[Owned villa]	1.09	0.82–1.43	0.567
BMI	0.92	0.87–0.96	0.001
BMI CAT [reference: normal weight]			
[Underweight]	0.53	0.37–0.76	0.001
[Overweight]	0.89	0.64–1.24	0.506
[Obese]	0.78	0.34–1.80	0.563
Smoking [reference: never]			
[Current smoker]	1.06	0.79–1.43	0.705
[Ex-smoker]	0.99	0.63–1.58	0.955
[Passive smoker]	0.82	0.58–1.18	0.286
Physical activity [reference: no physical activity]			
[mild to moderate]	1.93	1.61–2.32	<0.001
[moderate to vigorous]	2.68	2.10–3.44	<0.001
Eating Behavior: whole grain			
[Yes]	1.09	0.92–1.29	0.317
Eating Behavior: daily fruits and vegetables			
[Yes]	2.08	1.67–2.61	<0.001
Eating Behavior: low fat meat			
[Yes]	1.90	1.61–2.25	<0.001
Eating Behavior: avoidance high sugar food			
[Yes]	1.83	1.53–2.20	<0.001
Eating Behavior: low fat products			
[Yes]	2.02	1.67–2.46	<0.001
Observations	3411		
R^2^	0.16		

## Data Availability

The raw data supporting the conclusions of this article are available upon reasonable request to the corresponding author.
